# Presence of H3K4me3 on Paternally Expressed Genes of the Paternal Genome From Sperm to Implantation

**DOI:** 10.3389/fcell.2022.838684

**Published:** 2022-03-10

**Authors:** Teruhito Ishihara, Oliver W. Griffith, Shunsuke Suzuki, Marilyn B. Renfree

**Affiliations:** ^1^ School of BioSciences, The University of Melbourne, Melbourne, VIC, Australia; ^2^ Department of Biological Sciences, Macquarie University, Sydney, NSW, Australia; ^3^ Department of Agricultural and Life Sciences, Faculty of Agriculture, Shinshu University, Nagano, Japan

**Keywords:** genomic imprinting, histone modification, DNA methylation, DMR, H3K27me3

## Abstract

Genomic imprinting, parent-of-origin-specific gene expression, is controlled by differential epigenetic status of the parental chromosomes. While DNA methylation and suppressive histone modifications established during gametogenesis suppress imprinted genes on the inactive allele, how and when the expressed allele gains its active status is not clear. In this study, we asked whether the active histone-3 lysine-4 trimethylation (H3K4me3) marks remain at paternally expressed genes (PEGs) in sperm and embryos before and after fertilization using published data. Here we show that mouse sperm had the active H3K4me3 at more than half of known PEGs, and these genes were present even after fertilization. Using reciprocal cross data, we identified 13 new transient PEGs during zygotic genome activation. Next, we confirmed that the 12 out of the 13 new transient PEGs were associated with the paternal H3K4me3 in sperm. Nine out of the 12 genes were associated with the paternal H3K4me3 in zygotes. Our results show that paternal H3K4me3 marks escape inactivation during the histone-to-protamine transition that occurs during sperm maturation and are present in embryos from early zygotic stages up to implantation.

## Introduction

Genomic imprinting is regulated by differential epigenetic states between the parental alleles pretranscriptionally and controls development and maternal behavior in both eutherian and marsupial (therian) mammals (Renfree et al., 2009). This process evolved in the common ancestor of therian mammals and has not been found in other vertebrates. There are two major epigenetic mechanisms of genomic imprinting: differential DNA methylation and differential histone modifications. Differential DNA methylation was the first mechanism proposed for controlling genomic imprinting (Li et al., 1993). DNA methylation (the addition of 5-methylcytosine; 5mC) at promoter cytosine–guanine-rich sites (CpG islands; CGI) normally suppresses gene expression (Bird and Wolffe, 1999), so that the allele with DNA methylation is inactive. Histone modifications define active or inactive chromatin status (Gates et al., 2017). Many DNA methylation-independent imprinted genes also present in eutherian mammals (Inoue et al., 2017a; Nagano et al., 2008; Wagschal et al., 2008). Characterization of the mechanisms of differential histone modification-based imprinting could shed light on the understanding of the evolution of genomic imprinting.

Since genomic imprinting is defined by the differences in epigenetic status between parental alleles, understanding how sex-specific epigenetic information is established and maintained in the genome of gametes is critical to elucidate imprinting mechanisms. The current model of genomic imprinting is based on studies focused on transmission of suppressive modifications including DNA methylation or histone-3 lysine-27 tri-methylation (H3K27me3). The active histone-3 lysine-4 tri-methylation (H3K4me3) is found preferentially enriched on the promoter of the unmethylated allele at imprinted genes in mouse embryonic stem cells (Mikkelsen et al., 2007) and mouse embryonic fibroblast cells (Singh et al., 2011; Andergassen et al., 2015). This suggests that preferential enrichment of H3K4me3 status on the un-methylated allele may help define the active chromatin status of the expressed allele. If paternally expressed genes (PEGs) are to be expressed, the paternally derived genome needs to have active chromatin status. One possible way to establish this is by transmission of paternal active histone modifications, such as H3K4me3, in sperm. However, most paternal histones are not retained in mature sperm. To tightly package the genome, histones of male germ cells are replaced by another protein, protamine, to condense sperm DNA in the last phase of spermatogenesis. This is known as the histone-to-protamine transition (McLay and Clarke, 2003). Transfer of paternal-specific histone information would not occur if all the histones were converted to protamines, which are later replaced by maternally supplied histones after fertilization (McLay and Clarke, 2003). Approximately 90% of histones are changed into protamines by the histone-to-protamine transition in humans and 99% in mice (Balhorn et al., 1977; Gatewood et al., 1987). Although there is a massive loss of histone information in sperm, human sperm do retain histones with H3K4me3 at a subset of the PEGs, such as *KCNQ1OT1*, *MEST*, and *PEG10* (Hammoud et al., 2009). In mouse sperm, *Kcnq1ot1* also retains H3K4me3 on its promoter after the histone-to-protamine transition (Delaval et al., 2007). These examples suggest that paternal H3K4me3 information might be retained more widely in eutherian sperm.

Although paternally transmitted histone information is present at the subset of PEGs in sperm, the extent to which histone transmission may be associated with genomic imprinting after fertilization is still not defined. We hypothesized that paternally active chromatin with H3K4me3 remains in active sperm throughout the histone-to-protamine transition and even after fertilization provides active epigenetic status of PEGs during paternal genome activation in mouse preimplantation embryos. To address this hypothesis, publicly available mouse sequencing datasets were analyzed (see *Methods* section). Mouse round spermatids and sperm ChIP-seq data sets were analyzed to identify PEGs retaining the active H3K4me3 marks after the histone-to-protamine transition. Parental histone modifications were tracked up to the blastocyst stage to confirm retention of the paternal active status during embryogenesis. Combining ChIP-seq data with RNA-seq data from pre-implantation embryos derived from reciprocal crosses, we identified eight new transient PEGs associated with the H3K4me3 on paternal genome before and after fertilization. We suggest that H3K4me3 marks are retained by paternal histone transmission, correlating with the active status of PEGs after conception and up to implantation.

## Materials and methods

### Datasets

Publicly available raw sequencing datasets were downloaded from either NCBI SRA (RRID:SCR_004891, https://www.ncbi.nlm.nih.gov/sra) or DDBJ SRA (RRID:SCR_001370, https://www.ddbj.nig.ac.jp/dra/index-e.html). All ChIP-seq, RNA-seq, and WGBS data used in this study is summarized in Supplementary Table S1. Round spermatid H3K4me3 and H3K27me3 ChIP-seq datasets were obtained from GSE49624 (Hammoud et al., 2014). Sperm H3K4me3 ChIP-seq data were obtained from DRX117177 (Yamaguchi et al., 2018). Sperm H3K4me3 and H3K27me3 ChIP-seq datasets were obtained from GSE42629 (Erkek et al., 2013). RNA-seq and H3K4me3 ChIP-seq data of oocyte, sperm, PN3-zygote, PN5-zygote, two-cell, four-cell, eight-cell embryos, and blastocyst derived from crosses PWK/PhJ (Father) x C57BL/6N (Mother) were from GSE71434 (Zhang et al., 2016). RNA-seq data of two- and four-cell embryos derived from reciprocal crosses PWK/PhJ (Father) x C57BL/6N (Mother) and C57BL/6N (Father) x PWK/PhJ (Mother) were obtained from GSE127106. RNA-seq data of androgenotes (two-cell stage), parthenogenotes (two-cell stage), amanitin-treated two-cell embryos, and placenta tissues derived from reciprocal crosses PWK (Father) x B6GFP (Mother) and B6GFP (Father) x PWK (Mother) were obtained from GSE92605 (Inoue et al., 2017a). WGBS data of MII oocytes were obtained from GSE56879 (Smallwood et al., 2014). H3K27me3 ChIP-seq data of sperm and MII oocyte were obtained from GSE76687 (Zheng et al., 2016).

### Mouse sperm and round spermatid ChIP-seq data analysis

To confirm active histone retention at the known PEGs throughout the histone-to-protamine transition in mice, ChIP-seq data using mouse sperm and round spermatids were analyzed (Supplementary Table S1). All ChIP-seq reads were trimmed using TrimGalore! (v0.6.5, https://github.com/FelixKrueger/TrimGalore) with default settings to remove adaptor sequences, low-quality reads, and less than 20-bp reads. The trimmed reads were aligned to the mouse reference genome (mm10) using Bowtie2 (v2.3.4.3, RRID:SCR_016368) (Langmead and Salzberg, 2012). PCR duplicates were filtered by Picard tool MarkDuplicates (v2.25.0, RRID:SCR_006525), and low mapping quality reads (MAPQ <30) including nonmapped reads were removed by Samtools (v1.9, RRID:SCR_002105). After the filtration, the mapped reads were used for peak calling by comparing it with input control using MACS2 (v2.1.3.3, RRID:SCR_013291) (Zhang et al., 2008) with the parameters -f BAMPE --broad --broad-cutoff 0.05 for paired-end reads and --broad --broad-cutoff 0.05 --nomodel for single-end reads. The mapped reads from datasets without input control were also used for peak calling using MACS2 with the parameters --broad -f BAMPE --broad-cutoff 0.05 --nolambda --keep-dup auto for paired-end reads and --broad --broad-cutoff 0.05 --nolambda --keep-dup auto --nomodel for single-end reads. The called peaks and associated genes were annotated with ChIPpeakAnno (v3.6.5, RRID:SCR_012828) (Zhu et al., 2010). After comparing different datasets, PEGs common to more than one dataset were considered as the histone modification-associated PEGs in sperm. Bedgraph coverage tracks were generated from aligned reads using Deeptools (v3.3.1, RRID:SCR_016366) (Ramírez et al., 2016). The coverage was calculated as the number of reads per 10-bp bins across the genome and normalized using reads per kilobase per million (RPKM) mapped reads. Information about known paternally expressed genes was downloaded from http://www.geneimprint.com/site/genes-by-species.Mus+musculus and https://www.mousebook.org/imprinting-gene-list (MouseBook, RRID:SCR_006358). Peaks of H3K4me3 and H3K27me3 at the known PEGs were visualized with MACS2 output using Spark (v2.6.2) (Kurtenbach and Harbour, 2019). Information about CpG islands of the repeat unmasked mouse genome was obtained from UCSC Genome Browser (RRID:SCR_005780, https://genome.ucsc.edu).

### Allelic analysis of histone-3 lysine-4 trimethylation during mouse embryogenesis using public ChIP-seq data

To track the retained histone information at the PEGs after fertilization, ChIP-seq data using mouse sperm, oocytes, and embryos were analyzed (Supplementary Table S1). All ChIP-seq reads were trimmed using TrimGalore! (v0.6.5, https://github.com/FelixKrueger/TrimGalore) with default settings. The trimmed reads of MII oocytes, maternal pronucleus, and paternal pronucleus of a pronuclear stage 3 (PN3) zygote were aligned to the mouse reference genome (mm10) using Bowtie2 (v2.3.4.3) (Langmead and Salzberg, 2012) with default settings. PCR duplicates were filtered by Picard tool MarkDuplicates (v2.25.0, RRID:SCR_006525), and low mapping quality reads (MAPQ <30) including nonmapped reads were removed by Samtools (v1.9, RRID:SCR_002105). After the filtration, the mapped reads were used for peak calling using MACS2 (v2.1.3.3) (Zhang et al., 2008) with the parameters -f BAMPE --broad --keep-dup auto --broad-cutoff 0.05 --nolambda to identify regions having a significant H3K4me3 peak. Information about promoter regions of known PEGs are derived from the Ensemble website (http://asia.ensembl.org/index.html). As for early embryos from PN5-stage zygotes to inner cell mass (ICM), the trimmed reads were aligned to mm10 with Bowtie2 followed by assigning reads to each parental allele by SnpSplit (v0.3.4) (Krueger and Andrews, 2016). Before mapping reads to the mouse genome (mm10), all SNP sites were N-masked with SnpSplit (v0.3.4) based on SNP information of the PWK/PhJ strain and the C57BL/6N strain from the Sanger Institute (http://www.sanger.ac.uk/science/data/mouse-genomes-project). Thereafter, the trimmed reads of early embryos were aligned to the N-masked mouse reference genome using Bowtie2 with a parameter --end-to-end to avoid soft clipping. The mapped reads were then split into either the PWK/PhJ allele or the C57BL/6N allele by SnpSplit (v0.3.4). As the two replicates were highly correlated to each other (Pearson’s correlation >0.96), the two replicates were then pooled together for each stage for the downstream analysis. Bedgraph coverage tracks were generated from the allele-splitted reads using Deeptools (v3.3.1) (Ramírez et al., 2016). The allelic coverage was calculated separately as the number of allele-assigned reads per 10-bp bins across the genome and normalized using RPKM. To calculate the allelic ratio of ChIP-seq reads, PCR duplicates were removed by Picard (v2.25.0, RRID:SCR_006525), and the deduplicated allelic reads at around promoters of imprinted genes were counted. Before counting allelic reads, H3K4me3 peaks around promoters of imprinted genes were called using mapped reads before allele assigning in ICM by MACS2 with the parameters --broad --nolambda --keep-dup all --broad-cutoff 0.01 --nomodel. The MACS2 output from ICM and CpG island information on IGV-web (https://igv.org/app/) were combined to define regions including promoter CpG islands for counting reads by FeatureCount (v2.0.1) (Liao et al., 2014). Thereafter paternal allelic ratio was calculated by the following equation: paternal allelic ratio (%) = (paternal allele specific reads/sum of both paternal and maternal allelic reads) × 100. To estimate general allelic imbalance of raw reads at each stage, allelic ratios of total paternal allele-specific reads against the sum of each allele-assigned reads were calculated. By considering the ratio as an expected allelic ratio of each stage, the chi-square test was performed to test paternal enrichment of the counted allelic reads at each gene using R. Loss of paternal enrichment of H3K4me3 at PEGs was tested by the two-sided Cochran-Armitage trend test in R.

### Allelic expression analysis using mouse RNA-seq data

To identify H3K4me3 transmission associated-paternally expressed genes in mouse early embryos, RNA-seq data using mouse embryos were analyzed (Supplementary Table S1) and combined with the MACS2 output. All RNA-seq reads were trimmed using TrimGalore! (v0.6.5) (https://github.com/FelixKrueger/TrimGalore) with default settings. Before mapping reads to the mouse genome, all SNP sites were N-masked with SnpSplit (v0.3.4) (Krueger and Andrews, 2016) based on SNP information of the PWK/PhJ strain and the C57BL/6N strain from the Sanger Institute (http://www.sanger.ac.uk/science/data/mouse-genomes-project). Thereafter, the trimmed reads were aligned to the N-masked mouse reference genome using HISAT2 (v2.1.0, RRID:SCR_015530) (Kim et al., 2019) with parameters --sp 1000,1000 to avoid soft clipping. The mapped reads were then split into the PWK/PhJ allele or the C57BL/6N allele by SnpSplit (v0.3.4). As mouse zygotic genome activation occurs in two-cell embryos (Fraser and Lin, 2016), RNA-seq data sets (early two-cell, late two-cell and four-cell, cell-cell embryos) derived from a cross, PWK/PhJ (Father) x C57BL/6N (Mother), were initially analyzed to characterize the imprinted gene expression of the known PEGs. The assigned reads of each biological replicate of each stage were then passed to HTSeq (v0.11.2, RRID:SCR_005514) (Anders et al., 2015) with parameters -r pos a to estimate the expression levels of each gene. Thereafter, all the transcripts and abundance from HTSeq were analyzed by edgeR (v1.22.2, RRID:SCR_012802) (Robinson et al., 2009) based on the parental alleles and two biological replicates, and then determined which genes were differentially expressed between the parental alleles across the biological replicates of each stage. To identify new imprinted genes during zygotic genome activation, RNA-seq data sets (early two-cell, late two-cell, and four-cell embryos) derived from reciprocal crosses, PWK/PhJ (Father) x C57BL/6N (Mother) and C57BL/6N (Father) x PWK/PhJ (Mother), were chosen for the downstream allelic expression analysis (Supplementary Table S1). The assigned reads of each biological replicate of each stage were also passed to HTSeq (v0.11.2) with parameters -r pos to estimate the expression levels of each gene. All the transcripts and abundance from HTSeq were analyzed by edgeR (v1.22.2) as multivariate analysis based on the reciprocal crosses, the parental alleles, and two biological replicates, and then which genes were differentially expressed between the parental alleles across the biological replicates of each stage were determined. Genes having log_2_FC > 3 and FDR <0.001 were considered as PEG candidates. Those genes identified as PEG candidates were also analyzed using a different dataset derived from a cross, PWK/PhJ (Father) x C57BL/6N (Mother), to confirm its paternal expression. If the PEG candidates also had log_2_FC >3 and FDR <0.001 in the data from the second study, the genes were considered as PEGs. The PEGs were further examined using RNA-seq data of two-cell embryos derived from androgenotes, parthenogenotes, and amanitin-treated embryos (Supplementary Table S1). All RNA-seq reads were trimmed using TrimGalore! (v0.6.5) (https://github.com/FelixKrueger/TrimGalore) with default settings. The trimmed reads were mapped against the mouse reference genome (mm10) with default settings. The mapped reads of each biological replicate of each group (androgenotes, parthenogenotes, and amanitin treated) were then passed to HTSeq (v0.11.2, RRID:SCR_005514) (Anders et al., 2015) to estimate the expression levels of each gene. Thereafter, all the transcripts and abundance from HTSeq were analyzed by edgeR (v1.22.2, RRID:SCR_012802) (Robinson et al., 2009) based on the groups and two biological replicates, and then which genes were differentially expressed between the parental alleles across the biological replicates of each group were determined. RNA-seq data of two-cell embryos with amanitin treated were used as controls for the absence of major zygotic genome activation. After comparing with androgenotes and parthenogenotes, genes having log_2_FC >1 were considered as PEGs. Potential *cis*-regulatory elements, such as endogenous retroviral motifs, were searched with RepeatMasker on the UCSC genome browser (https://genome.ucsc.edu).

### Mouse methylome analysis using whole-genome bisulfite-seq data

To characterize the mouse methylome before and after fertilization, whole-genome bisulfite sequencing (WGBS) data using mouse oocytes and embryos were analyzed (Supplementary Table S1). Raw WGBS reads were trimmed to remove both poor-quality reads and adapter sequences using TrimGalore! (v0.6.5) (https://github.com/FelixKrueger/TrimGalore) with parameters --clip_r1 9 --clip_r2 9 --paired. The trimmed paired-end reads were separately aligned to the mouse genome as single-end reads with Bismark (v0.22.3, RRID:SCR_005604) (Krueger and Andrews, 2011) with the parameters --bowtie2 --non_directional to maximize the mapping ratio. Aligned reads were then merged and de-duplicated with de-duplicate_bismark function in Bismark (v0.22.3). CpG methylation was specifically called from the deduplicated output using Bismark_methylation_extractor function in Bismark (v0.22.3) with the parameters --bedGraph --report. Mouse sperm methylome data were downloaded from NODAI genome research center (http://www.nodai-genome.org/mouse.html?lang=en) (Kobayashi et al., 2012). CpG methylation levels were visualized with R. Information about CpG islands of repeat unmasked mouse genome was obtained from UCSC Genome Browser (https://genome.ucsc.edu).

## Results

### More than half of known paternally expressed genes retained active histone-3 lysine-4 trimethylation marks in mouse sperm throughout the histone-to-protamine transition

**FIGURE 1:**
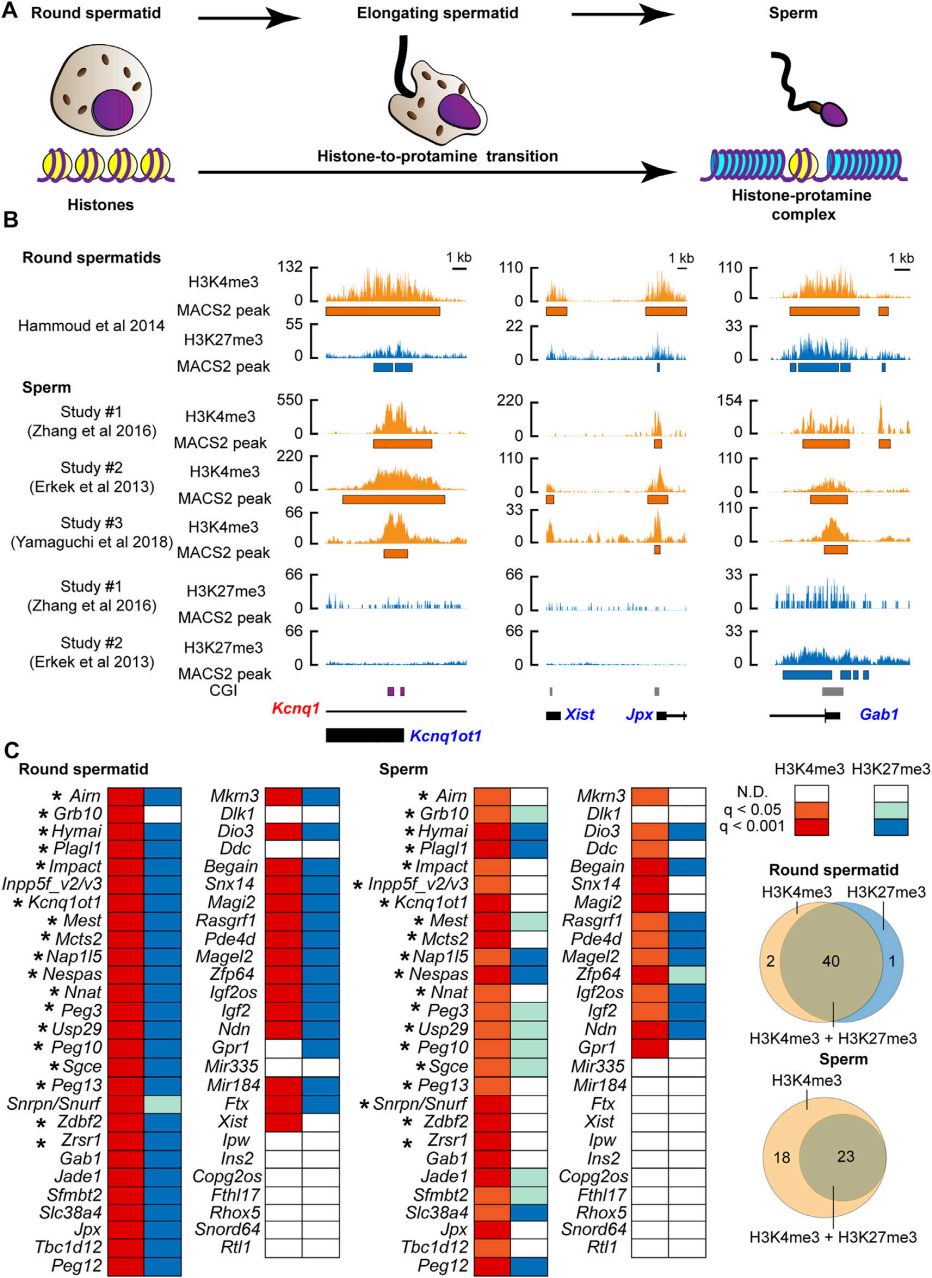
The active histone-3 lysine-4 trimethylation (H3K4me3) marks were retained at known paternally expressed genes in mouse sperm. **(A)** Schematic model of the histone-to-protamine transition during the final phase of spermatogenesis. **(B)** Examples of the active H3K4me3 and the suppressive H3K4me3 status before and after the histone-to-protamine transition. *Kcnq1ot1* is shown as an example of the known gDMR-dependent imprinted gene, and *Jpx* and *Gab1* are shown as examples of the known DMR-independent imprinted genes. CpG island (CGI) where the germline DMR is present is shown by the purple box. Orange shows H3K4me3, and aqua shows H3K27me3 coverage tracks. Annotated exon information is under the coverage tracks with black-colored boxes and lines. Allelic expressions of transcripts are represented by blue (paternal) and red (maternal) letters. The orange and aqua-colored boxes under the coverage tracks represent regions of statistically significant peaks based on MACS2. **(C)** H3K4me3 and H3K27me3 status during the histone-to-protamine transition. After comparing more than two datasets, PEGs common including *Inpp5f_v2/v3* and *Snrpn/Snurf* to more than one data set were considered as the histone modification-associated PEGs in sperm. Asterisks represent genes with H3K4me3 at the known gDMRs. The q-values are based on MACS2 peak calling, and the q-value color is based on the highest q-value across datasets. The Venn diagrams show the number of genes associated with either H3K4me3 or H3K27me3.

We asked if changes in histone information at PEGs occurred during the histone-to-protamine transition. We examined the active (H3K4me3) and inactive (H3K27me3) chromatin status using publicly available ChIP-seq datasets of mouse round spermatids (before the transition: Figure 1A) (Hammoud et al., 2014) and sperm [after the transition: Figure 1A) (Erkek et al., 2013; Zhang et al., 2016; Yamaguchi et al., 2018) (Supplementary Table S1). As a result of peak calling, in round spermatids, the majority of known PEGs (39 genes out of 53 genes listed in the two online databases (http://www.geneimprint.com/site/genes-by-species.Mus+musculus and https://www.mousebook.org/imprinting-gene-list)] were confirmed to have not only the active H3K4me3 but also the suppressive H3K27me3 marks (Figures 1B,C). This included four known H3K27me3-dependent imprinted genes, *Sfmbt2*, *Slc38a4*, *Gab1*, and *Jade* (Inoue et al., 2017a). After comparing the sperm datasets, PEGs common to more than one dataset were considered as the histone modification-associated PEGs in sperm. As a result, after the histone-to-protamine transition, 41 out of the 53 known PEGs were identified as H3K4me3-associated genes in sperm (Figures 1B,C and Supplementary Figure S1). Although some genes lacked the inactive H3K27me3, more than half of these genes also retained the H3K27me3 (Figures 1B,C and Supplementary Figure S1). Within the 41 genes, 17 regions (20 genes) retaining the H3K4me3 active modification were known germline differentially methylated regions (gDMRs) including the *Kcnq1ot1* gene locus (Hiura et al., 2006; Ruf et al., 2007; Wood et al., 2007; Wiley et al., 2008; Williamson et al., 2011; Iglesias-Platas et al., 2012) (Figures 1B, C). A known H3K27me3-dependent imprinted gene, *Xist* (Inoue et al., 2017b), had the active H3K4me3 marks in round spermatid but lacked significantly enriched active marks in the two sperm H3K4me3 datasets analyzed in this study (Figures 1B,C and Supplementary Figure S1). In contrast to this, *Jpx*, an activator of *Xist* (Tian et al., 2010), had both the active H3K4me3 and inactive H3K27me3 marks in round spermatids, but only the active H3K4me3 marks were significantly enriched in all sperm H3K4me3 datasets analyzed in this study (Figures 1B, C and Supplementary Figure S1). Three known H3K27me3-dependent imprinted genes, *Jade1*, *Slc38a4*, and *Sfmbt2*, had both H3K4me3 and H3K27me3 in round spermatids and retained both in sperm (Figure 1C and Supplementary Figure S1). A known H3K27me3-dependent imprinted gene, *Gab1*, also had both H3K4me3 and H3K27me3 in round spermatids, and there was a significantly enriched H3K4me3 in sperm. Although significant H3K27me3 was not present at *Gab1* in one of the datasets, the other showed significant H3K27me3 at the promoter of *Gab1* in sperm (Figures 1B,C and Supplementary Figure S1. Thus, sperm H3K27me3 status at some of PEGs, such as *Gab1*, was varied in datasets. However, histone retention with the active H3K4me3 and the suppressive H3K27me3 at PEGs appears to be widespread for PEGs in the sperm, regardless of their maternal imprinting status (either H3K27me3 or gDMR).

### Active epigenetic status presented in sperm were maintained during early embryogenesis

**FIGURE 2:**
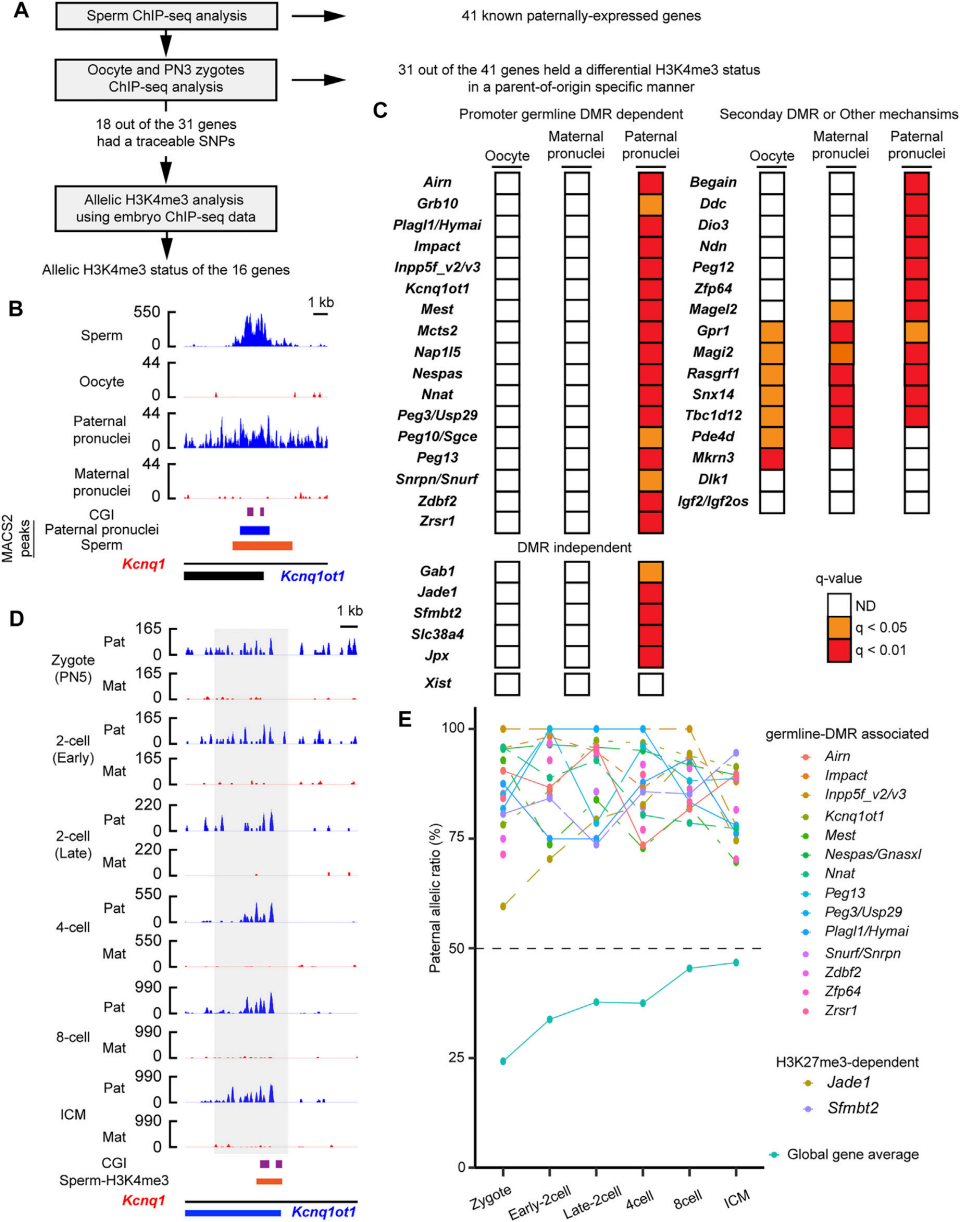
Paternal active chromatin status was retained during embryogenesis. **(A)** Schematic workflow of the characterization of paternal H3K4me3 status at the known paternally expressed genes (PEGs) during mouse embryogenesis. **(B)** H3K4me3 status before and just after fertilization of a known PEG, *Kcnq1ot1*. gDMR is shown by the purple boxes. Blue-colored boxes: significant H3K4me3 peaks in the paternal pronuclei. The orange-colored box: H3K4me3-retained region in sperm defined by MACS2. **(C)** H3K4me3 status of known PEGs in oocytes and PN3 zygote. Promoter gDMR-dependent and DMR-independent PEGs hold differential H3K4me3 status in a parent-of-origin-specific manner in the PN3 zygote. In contrast, known secondary DMR-dependent imprinted genes, such as *Mkrn3* and *Igf2os*, lacked a differential H3K4me3 status in the PN3 zygote. **(D)** An example of promoter gDMR-dependent imprinted gene, which retained paternal enrichment of H3K4me3 during embryogenesis. The allelic coverage tracks: blue (paternal) and red (maternal), respectively. The *y*-axis of each coverage track is the reads per kilobase per million (RPKM) values. The region where counting reads was performed is enclosed by the gray-colored box. gDMR is shown by the purple box. The orange-colored box represents the H3K4me3-retained region in sperm defined by MACS2. **(E)** Paternal enrichment of H3K4me3 at the promoters of 18 genes is maintained in the developing mouse embryos. Allelic ratio of total paternal allele-specific reads against total raw reads are shown as “global gene average.” ICM, inner cell mass.

Although histone information is dynamically reprogrammed due to the global wave of epigenetic reprogramming during early embryogenesis (Zhang et al., 2016; Zheng et al., 2016; Xia et al., 2019), the paternal H3K4me3 but not the H3K27me3 escapes the reprogramming (Lismer et al., 2020). To track whether the paternal active histone information was retained at the PEGs after fertilization, the parental H3K4me3 enrichment was tracked by analysis of published ChIP-seq data of mouse embryos and oocytes (Zhang et al., 2016) (Figure 2A and Supplementary Table S1). At the pronuclear stage 3 (PN3) zygotes, the paternal pronuclei had significant H3K4me3 peaks at the 34 regions (37 genes), of which 28 regions (31 genes) lacked H3K4me3 peaks in the maternal pronuclei (Figures 2B,C). The 28 regions included the 17-known gDMR (Figures 2B,C) and the five DNA methylation-independent imprinted genes, *Jpx*, *Gab1*, *Jade1*, *Slc38a4*, and *Sfmbtl* (Figure 2C). Using mouse oocyte ChIP-seq data, the 28 regions (31 genes) were confirmed to lack significant H3K4me3 peaks (Figure 2C), showing that the H3K4me3 histone marks at the 31 genes in the PN3 zygotes held a differential H3K4me3 status in a parent-of-origin-specific manner. In contrast to this, known secondary DMR-associated imprinted genes, such as *Mkrn3* and *Igf2os*, lacked the paternal-specific H3K4me3 modification at their promoter CpG islands (CGIs) in the paternal pronucleus (Figure 2C). We also examined parental H3K4me3 status at the H3K27me3-dependent imprinted gene, *Xis*t, and confirmed that there was no significant H3K4me3 at the promoter of *Xist*. Sixteen regions out of the 28 regions (18 genes out of the 31 PEGs) had a traceable parental SNP information around the histone-retained regions in sperm. Promoters of 16 out of the 18 genes showed paternal enrichment of the active H3K4me3 modification at all stages up to the blastocyst stage (*p* < 0.05, chi-square test) (Figures 2D,E). PEGs, *Plagl1* and *Hymai*, also showed paternal enrichment at most developmental stages (*p* < 0.05, chi-square test) except at the early two-cell stage (*p* = 0.082, chi-square test). At least 16 known PEGs including the two DNA methylation-independent imprinted genes, *Jade1 and Sfmbt2*, retained the paternally skewed H3K4me3 status throughout the global wave of epigenetic reprogramming during early embryogenesis.

### Eight out of 16 known paternally expressed genes initiated imprinted gene expression during zygotic genome activation

**FIGURE 3:**
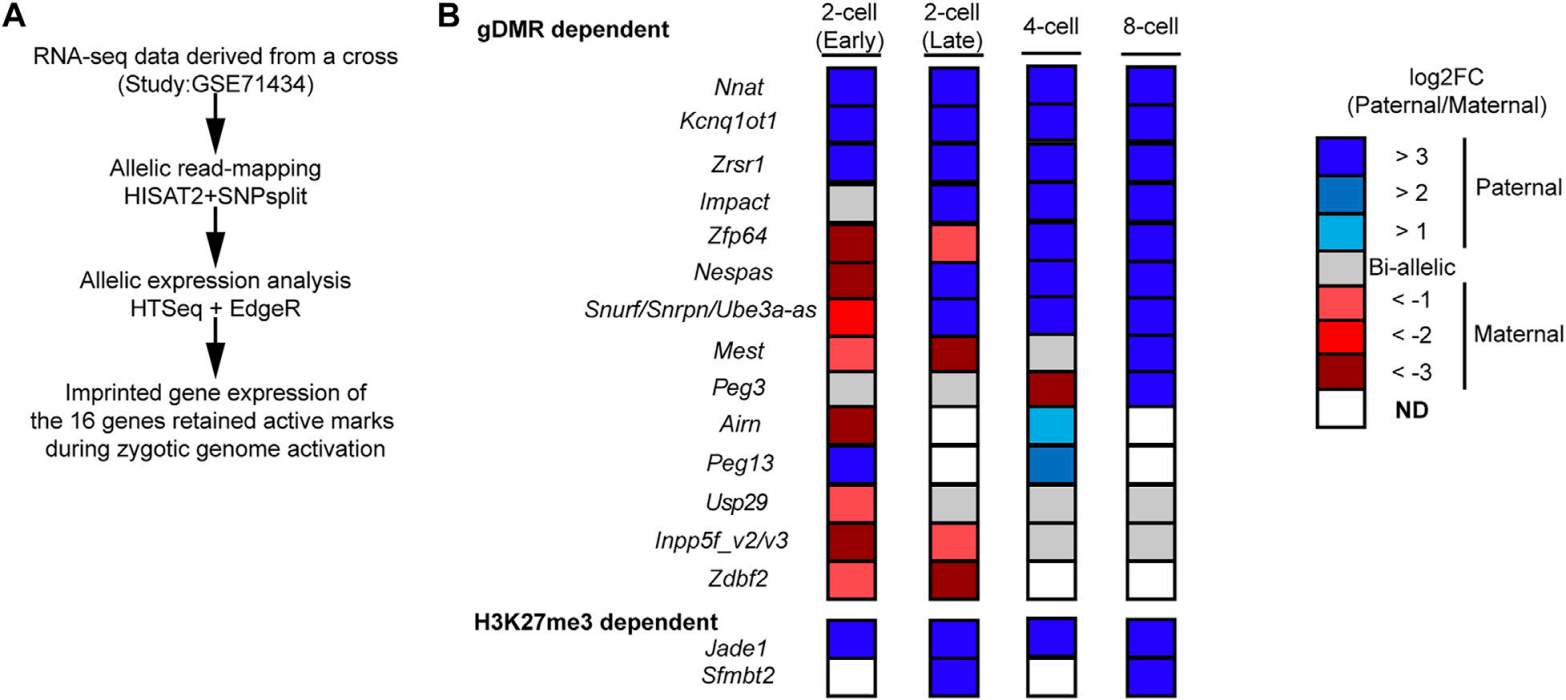
Majority of the 16 PEGs began imprinted gene expression during zygotic genome activation. **(A)** Schematic workflow of allelic expression analysis of the 12 genes, which hold paternal H3K4me3 enrichment up to the blastocyst stage, during zygotic genome activation. **(B)** Allelic expression of the 16 imprinted genes during zygotic genome activation. Most of the genes began paternal expression during zygotic genome activation.

If the paternal enrichment of H3K4me3 at the 16 genes regulates their expression, it is possible that the 16 genes initiate their imprinted expression during paternal genome activation. Mouse major zygotic genome activation begins around the two-cell stage (Fraser and Lin, 2016), so RNA-seq data sets (early two-cell, late two-cell and f-cell, 8-cell) derived from a cross, PWK/PhJ (Father) x C57BL/6N (Mother) (Zhang et al., 2016) (Supplementary Table S1), were initially analyzed to characterize the imprinted gene expression of the 16 known PEGs (Figure 3A). In the early two-cell stage, five genes, *Nnat*, *Kcnq1ot1*, *Zrsr1*, *Peg13*, and *Jade1* showed paternal expression (Figure 3B). In the late two-cell stage, an additional four genes, *Impact*, *Snrpn/Snruf/Ube3a-as*, *Nespas*, and *Sfmbt2*, began paternal expression (Figure 3B). In the four-cell stage, *Zfp64* initiated its paternal expression, and *Airn* and *Peg13* showed weak paternal expression (Figure 3B). Due to the presence of a non-imprinted isoform, allelic expression of the isoform-dependent imprinted gene, *Inpp5fv2*/*Inpp5fv3*, could not be confirmed. Although some genes did not consistently show paternal expression, up to the eight-cell stage, 10 genes and *Snrpn/Snruf/Ube3a-as* began paternal expression (Figure 3B).

### Eighteen out of 22 paternally expressed genes during zygotic genome activation retained paternal histone information in sperm

**FIGURE 4:**
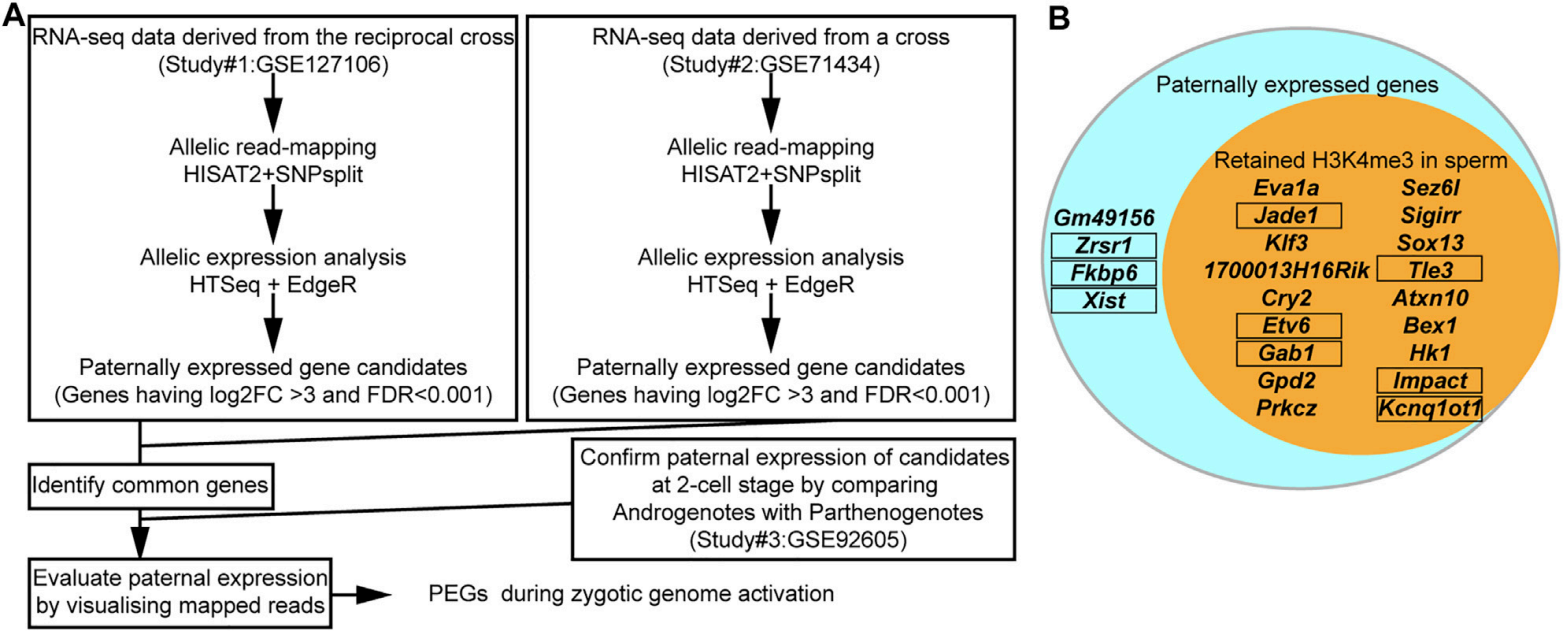
The majority of PEGs during zygotic genome activation had H3K4me3 in sperm. **(A)** Schematic workflow of the identification of PEGs. **(B)** Most identified PEGs hold the active H3K4me3 marks in sperm. Aqua circle: all 22 identified PEGs during zygotic genome activation. Orange circle: 18 genes with sperm H3K4me3. Known PEGs enclosed by black framed boxes.

Eight out of the 16 known PEGs showed paternal expression during the major zygotic genome activation (from the early two- to four-cell stage). It is, therefore, possible that there are many more imprinted genes that also have similar paternal expression during paternal genome activation. To identify PEG candidates during zygotic genome activation, RNA-seq data (from early two-cell, late two-cell, and four-cell embryos) derived from a reciprocal cross, PWK/PhJ (Father) x C57BL/6N (Mother) and C57BL/6N (Father) x PWK/PhJ (Mother), were analyzed (Supplementary Table S1). After comparing the two different datasets derived from two different studies (Figure 4A and Supplementary Table S1), a total of 22 genes were confirmed as common PEGs during zygotic genome activation, of which 13 were newly described PEGs (Table 1). We further evaluated these newly described PEGs by performing differential expression analysis using RNA-seq data of two-cell embryos from androgenotes, parthenogenotes, and amanitin-treated embryos (Figure 4A and Supplementary Table S1). The amanitin-treated embryos were used to estimate expression of the 13 genes before zygotic genome activation. By comparing androgenotes with parthenogenotes, we confirmed that 11 out of 13 genes more than doubled expression in androgenotes compared with parthenogenotes (Log_2_FC >1, FDR <0.1). The other two genes showed biallelic expression (Log_2_FC <1). One of the two genes, *Gpd2*, was present before zygotic genome activation based on the amanitin-treated two-cell embryos. It was biallelically expressed (FDR ∼0.1) due to the presence of transcript before zygotic genome activation. Although there was almost no expression in the amanitin-treated two-cell embryos, the other gene, *Prkcz*, also had biallelic expression (FDR ∼0.5). As the high FDR and this gene showed paternal expression after the late two-cell stage in our analysis, we could not conclude its paternal expression based on the differential expression analysis between androgenotes and parthenogenotes.

**TABLE 1:** Summary of newly identified transient paternally expressed genes (PEGs) in this study.

Gene name	Association with H3K4me3 in sperm	Association with paternal H3K4me3 in PN3 zygotes	Having traceable SNPs at the promoter/H3K4me3-enriched region
*Atxn10*	Present	Present	Present
*Bex1*	Present		
*Cry2*	Present	Present	Present
*Eva1a*	Present	Present	Present
*Gm49156*	—		
*Gpd2*	Present	Present	Present
*Hk1*	Present		
*Klf3*	Present	Present	Present
*Sez6l*	Present	Present	
*Sigirr*	Present	Present	Present
*Sox13*	Present	Present	
*Prkcz*	Present	Present	Present
*1700013H16Rik*	Present		
Total number (13)	12	9	7

After comparing with the MACS2 output from the sperm ChIP-seq analysis, 18 out of the 22 identified PEGs (approximately 82%) were confirmed to have retained H3K4me3 in sperm (Figure 4B). The six-known PEGs, *Kcnq1ot1*, *Impact*, *Gab1*, *Jade1*, *Etv6*, and *Tle3*, were included in the 18 genes. So, the remaining 12 genes out of the 18 genes were newly described PEGs associated with the sperm H3K4me3 (Table 1).

Since early mammalian zygotic transcription includes repetitive elements including endogenous retroviruses (ERVs) (Schoorlemmer et al., 2014), it is possible that the newly described PEGs have ERV-derived *cis*-elements. Using UCSC genome browser, we asked whether common repetitive elements are present at promoters of the 13 newly described PEGs. As a result, seven out of the 13 new PEGs had ERV motifs within 2.5 kb from their transcription start sites. *Atxn10*, *Cry2*, and *Klf3* had ERVL-MaLR family motifs, *Prkcz*, *Sez6l*, and *Hk1* had ERVK family motifs, and *1700013H16Rik* had ERV1 motif.

As imprinting status is defined by allelic imbalances, suppressive modifications on the maternal allele are essential for the PEGs. *Jade*1, *Gab1*, *Etv6*, and *Tle3* are known to be associated with the maternal suppressive histone modification, H3K27me3 (Inoue et al., 2017a), so germline H3K27me3 as well as germline DNA methylation of the 18 genes associated with sperm H3K4me3 were investigated by analyzing published datasets (Kobayashi et al., 2012; Smallwood et al., 2014; Zheng et al., 2016) (Supplementary Table S1). Although the known germline DMR-associated imprinted genes lacked the H3K27me3 modification, the 12 newly identified PEGs showed enrichment of H3K27me3 modification on their promoter CGIs and gene bodies in oocytes (Supplementary Figure S2). This suggests that the new PEGs are programmed as inactive genes in oocytes. The known germline-DMR-associated imprinted genes, such as *Impact*, had high DNA methylation at the known germline-DMR in oocytes (Supplementary Figure S2). There was no obvious differential DNA methylation at the promoter CGIs in the majority of the new PEGs (Supplementary Figure S2).

### Loss of differential histone-3 lysine-4 trimethylation during embryogenesis coincided with loss of paternally skewed expression of the newly identified imprinted genes

**FIGURE 5:**
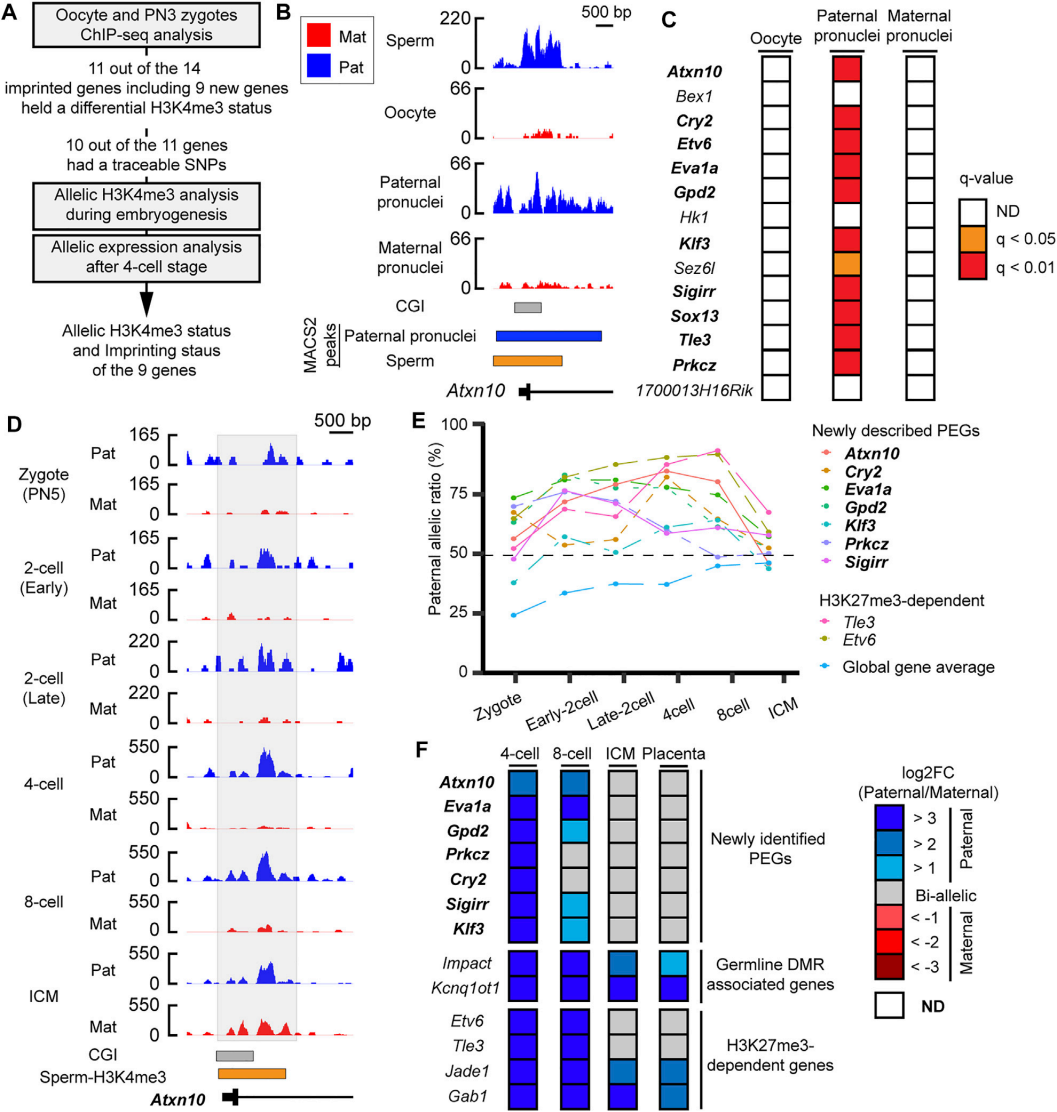
Differential H3K4me3 status of identified PEGs was reprogrammed as embryogenesis proceeded. **(A)** Schematic workflow of the characterization of paternal H3K4me3 status and allelic expression of the newly identified PEGs and the two known H3K27me3-dependent imprinted genes, *Etv6* and *Tle3* during mouse embryogenesis. **(B)** Active H3K4me3 status in gametes and zygote at a newly identified PEG, *Atxn10*. Lines with black boxes represent exon structures of the genes. Blue-colored boxes represent significant H3K4me3 peaks in the paternal pronuclei. The orange-colored box represents H3K4me3-retained region in sperm defined by MACS2. **(C)** H3K4me3 status of the 12 newly identified PEGs and the two known H3K27me3-dependent imprinted genes, *Etv6* and *Tle3*, in oocytes and PN3 zygote. As the paternal pronuclei have significant H3K4me3 peaks, but the maternal pronuclei lacked significant H3K4me3 peaks, 11 genes (bold text) including the two known H3K27me3-dependent imprinted genes, *Etv6* and *Tle3* (enclosed by black framed boxes), hold a differential H3K4me3 status in a parent-of-origin-specific manner. **(D)** H3K4me3 status after fertilization of a newly identified PEG, *Atxn10*. The allelic coverage tracks visualized by blue (paternal) and red (maternal), respectively. The *y*-axis of each coverage track represents RPKM values. The region where counting reads was performed is enclosed by the gray-colored box. **(E)** Paternal allelic ratios of H3K4me3 at the nine genes including seven newly identified PEG, which had SNPs, changed as embryogenesis proceeds. To overview general allelic imbalance of raw reads, paternal allelic ratio of total raw reads at each developmental stage are shown as “global gene average.” **(F)** Allelic expression of the newly identified PEG after the four-cell stage. New transient PEGs identified in this study are represented by the bold text. ICM, inner cell mass.

As most of the PEGs during zygotic genome activation retained H3K4me3 in the sperm, the paternal H3K4me3 may direct the active epigenetic status of these genes during zygotic genome activation. To characterize the paternal histone information in the newly identified PEGs during zygotic genome activation, paternal H3K4me3 information was tracked during early embryogenesis (Figure 5A). In the paternal pronuclei of the PN3 zygotes, 9 genes out of the 12 newly identified PEGs had significant H3K4me3 peaks around the histone-retained region, while the maternal pronuclei lacked the H3K4me3 peaks (Figures 5B,C and Table 1). The two known H3K27me3-dependent imprinted genes, *Etv6* and *Tle3*, also had significant H3K4me3 peaks around the histone-retained region, while the maternal pronuclei lacked the H3K4me3 peaks (Figure 5C). The ChIP-seq data analysis of oocyte showed that the H3K4me3 peaks were absent. Seven out of the nine newly identified PEGs and the two known H3K27me3-dependent imprinted genes, *Tle3* and *Etv6*, had informative SNPs around the H3K4me3 peaks (Table 1). Paternal H3K4me3 information at the region was traced up to the blastocyst stage. Paternal H3K4me3 retention during embryogenesis at the known PEGs, *Kcnq1ot1*, *Impact*, and *Jade1* was confirmed (Figure 2). In contrast to this, one of the new PEGs identified in this study, *Atxn10*, lost its paternally skewed H3K4me3 at the blastocyst stage (Figure 5D). Similarly, the other new PEGs identified in this study as well as two-known PEGs, *Etv6* and *Tle3*, lost their paternal-skewed H3K4me3 status as embryogenesis proceeded (*p* < 0.05, Cochran–Armitage trend test) (Figure 5E).

If the differential H3K4me3 status correlates with epigenetic status of the identified PEGs, the nine genes that showed loss of differential H3K4me3 may lose their imprinted gene expression as embryogenesis proceeds. To investigate imprinted gene expression of the nine PEGs after the four-cell stages, RNA-seq datasets using embryos derived from a cross, PWK/PhJ (Father) x C57BL/6N (Mother), were analyzed. Known PEGs such as *Impact* and *Kcnq1ot1* had paternal expression up to the blastocyst stage. However, all of the seven newly identified PEGs lost their imprinted gene expression as embryogenesis proceeded. All of the genes also showed biallelic expression in placental tissues derived from a reciprocal cross. Based on their transient allelic expression, we categorized them as transient PEGs. A newly identified transient PEG, *Prkcz*, lost differential H3K4me3 status at the eight-cell stage as the allelic ratio became about 49%. From the eight-cell stage, *Prkcz* showed biallelic expression. as Also, *Prkcz*, four newly identified transient PEGs, *Gpd2*, *Klf3*, *Sigirr*, and *Cry2*, began to lose paternally skewed expression when the paternal allelic ratio of H3K4me3 became about 60%. All of the genes lost imprinted gene expression coinciding with the developmental stages when the gene loci reprogrammed their differential H3K4me3 status between the parental alleles (Figures 5E,F).

## Discussion

Before the histone-to-protamine transition during sperm maturation, promoters of the majority of known PEGs had bivalent chromatin status marked by both H3K4me3 and H3K27me3. Even after the histone eviction, the active H3K4me3 and the inactive H3K27me3 were present at more than half of the known PEGs in the sperm. Since some of the PEGs only retained the active H3K4me3, retaining this modification in the sperm is a common feature of these PEGs, regardless of their known maternal imprinting status (either H3K27me3 or gDMR) in mice. Tracking the paternal histone information confirmed that at least 16 known PEGs with informative SNPs had paternal H3K4me3 enrichment during embryogenesis. By analyzing reciprocal crosses, the 13 new PEGs during zygotic genome activation were identified in this study. Twelve out of the 13 genes were associated with the paternal H3K4me3 in sperm, and 9 out of the 12 genes were associated with the paternal H3K4me3 in the PN3 zygote. By combining RNA-seq and ChIP-seq using parental SNP information, we confirmed that seven out of the nine genes lost their imprinted expression at different stages of early embryogenesis when differential H3K4me3 status was reprogrammed. Analyses of suppressive modifications including DNA methylation suggested that the majority of the transient PEGs are likely to be controlled by differential histone modification-based imprinting. By correlating the paternal H3K4me3 retention and the imprinting status of PEGs, our results suggest that histone retention with the active H3K4me3 in sperm may occur during the early stages of mouse genomic imprinting.

In both mouse and human sperm (Hammoud et al., 2009), not all PEGs retain active H3K4me3 modifications, suggesting that the transmission of H3K4me3 in sperm may not be the only mechanism for defining active status of PEGs. Eutherian imprinted genes tend to be clustered, and most of these clusters have been found to be under the control of imprinting centers. All of the maternal methylation-associated imprinting clusters (Arnaud, 2010) had active histone modifications at potential imprinting control centers in sperm. Some noncoding RNAs (ncRNAs) were expressed from imprinting center direct histone modifications to the neighboring genes (Royo and Cavaillé, 2008). One of the well-known imprinted gene clusters is a region on the X chromosome. There is a paternally expressed effector ncRNA, *Xist*, which acts as a *cis*-acting factor to inactivate the paternally inherited X chromosome (Brockdorff et al., 2020). *Xist* lacked the H3K4me3 modification in two of the three sperm H3K4me3 datasets analyzed in this study. In contrast to this, an activator of the ncRNA, *Jpx*, that induces *Xist* expression (Tian et al., 2010; Sun et al., 2013; Carmona et al., 2018) had the modification in the three datasets. So, the H3K4me3 retention at *Jpx* is more reliable, and the active modification at the activator gene may be enough to induce the active status of *Xist*. In fact, while *Jpx* retained the paternal H3K4me3 at PN3 zygotes, *Xist* lacked the paternal H3K4me3. So, if histone retention occurs at upstream genes, such as *Jpx*, not all PEGs need to retain active histone marks for histone retention to play a role in transmitting imprinting status. Another example is secondary DMR-associated genes. We confirmed that known secondary DMR-associated imprinted genes, such as *Mkrn3* and *Igf2os*, lacked the paternal specific H3K4me3 modification at their promoter CpG islands (CGIs) in the paternal pronucleus. The secondary DMRs are acquired during postimplantation development (Nechin et al., 2019), suggesting that the transmission of H3K4me3 is not necessary in establishing imprinting of those genes. As there are many active histone modifications, it is also possible that other modifications in sperm define the active status of some PEGs. Promoters of transcriptionally active genes are known to be associated with enriched lysine acetylation on histone H3 and H4 as well as H3K4me3 (Gates et al., 2017). However, hyper-acetylation occurs on the cores/tails of histones to facilitate the histone-to-protamine transition (Wang et al., 2019). The acetylated histones could facilitate histone eviction, so acetylation is less likely to be involved in genomic imprinting as transmittable paternal information, and there may be unknown transmittable histone modifications that also can define the active chromatin status in sperm.

Our results and results from human analyses (Hammoud et al., 2009) suggest that retaining the H3K4me3 active marks in sperm is a conserved feature of PEGs in human and mouse. However, it is still not clear how the imprinted gene loci retained the active H3K4me3 through the histone-to-protamine transition. Since the majority of PEGs were associated with both the active H3K4me3 and the inactive H3K27me3 before the histone-to-protamine transition, their promoter regions were transcriptionally inactive “bivalent” chromatin (Blanco et al., 2020). The bivalent chromatin is known to associate with many different protein complexes, such as Dppa2/4, polycomb repressive complex 2, and COMPASS (Lim and Meshorer, 2020). It is possible that the interaction with these proteins may help escape from the histone-to-protamine transition. The role of bivalent chromatin at the PEGs during the histone-to-protamine transition needs to be determined.

A subset of H3K4me3 alterations in sperm are retained and associated with alterations in gene expression in eight-cell embryos (Lismer et al., 2021). These data and our data suggest that the retained histone in sperm can be transmitted to the next generation. However, how the paternal active chromatin status is retained during embryogenesis is another important unanswered question. Using immunofluorescence, a previous study confirmed that there is a global absence/weakness of paternal H3K4me3 at the PN3 stage zygote, and detectable H3K4me3 emerges after the PN4 stage zygotes (Lepikhov and Walter, 2004). In contrast to this, we found that the regions where the H3K4me3 marks were present at most of the PEGs on the paternal pronucleus were consistent with the sperm H3K4me3-retained region. While only 1% of histones are retained in mouse sperm (Balhorn et al., 1977), oocytes acquire large H3K4me3 domains throughout oogenesis (Hanna et al., 2018). Therefore, the global paternal H3K4me3 weakness in immunofluorescence is possibly reflected in the gametic H3K4me3 imbalances. While the immunofluorescence study showed paternal H3K4me3 is present at the PN5 stage zygotes, Zhang et al. (2016) reported that paternal H3K4me3 is depleted at the zygote stage and re-established at the late two-cell stage. In that study, allele-specific H3K4me3 reads were normalized by calculating the differences of read numbers between both alleles (paternal–maternal) divided by the total number of allelic reads (materna + paternal). Therefore, this normalization method did not reflect the gametic H3K4me3 imbalances. However, taking the gametic H3K4me3 imbalances into account by using RPKM normalization on separate allelic reads showed paternal H3K4me3 retention at the PN5 stage zygotes (Lismer et al., 2021). We also confirmed paternal H3K4me3 retention at the 16 known PEGs even after the PN5 zygote. Similar statistically significant H3K4me3 enrichment on the paternal allele at eight imprinting control regions is observed in the developing mouse embryos (Zhang et al., 2016). As the paternal genome remodeling by oocyte-supplied histones occurs in the paternal pronucleus, current mechanisms do not explain how the active modifications are maintained at the PEGs after fertilization. Active chromatin status is difficult to transmit to daughter cells through the cell cycle (Escobar et al., 2019). Given that synthetic CG-rich sequences automatically acquire H3K4me3 (Wachter et al., 2014), it is possible that CG-rich sequences, like promoters of PEGs, automatically gain H3K4me3 as a default status. However, synthetic CG-rich sequences also gain H3K27me3 and form the transcriptionally inactive bivalent chromatin as a default setting (Wachter et al., 2014). We found that promoters of PEGs had the bivalent chromatin marked by H3K4me3 and H3K27me3 before and even after the histone-to-protamine transition. Moreover, loss of *Setd2*, which instructs DNA methylation at germline DMRs, causes aberrant H3K4me3 and H3K27me3 at the germline DMR of *Kcnq1ot1* in mouse oocyte (Xu et al., 2019). Therefore, promoters/ICRs of PEGs have the potential to recruit the suppressive H3K27me3 and lead to the establishment of transcriptionally inactive “bivalent” chromatin status (Blanco et al., 2020). However, after fertilization, at least eight known PEGs lack H3K27me3 on the paternal genome in zygotes (Inoue et al., 2017a), and sperm H3K27me3 at bivalent promoters is completely absent at the two-cell stage (Lismer et al., 2020). Collectively, these data suggest that there are unknown mechanisms underlying the maintenance of the active H3K4me3 chromatin status but not the H3K27me3 at the imprinting control regions on the paternal allele after fertilization. In mouse embryonic stem cells, H3K4me3 at bivalently marked promoters are catalyzed by mixed lineage leukemia 2 (Mll2) (Hu et al., 2013; Denissov et al., 2014). Mll2 can also bind to nonbivalent promoters. Since Mll2 deficiency in oocytes affects normal zygotic genome activation (Andreu-Vieyra et al., 2010), we speculate that Mll2 can associate with the paternal bivalent promoter at PEGs after fertilization to keep the paternally derived H3K4me3 information. To clarify this association between Mll2 and PEGs, the role of Mll2 on the paternal genome remodeling after fertilization needs to be examined.

There is an increasing awareness of the potential transgenerational effects of environmental toxicants throughout downstream modification of the sperm epigenome (Terashima et al., 2015; Ben Maamar et al., 2018; Skinner et al., 2018). In this context, histone marks in sperm that escape reprogramming are of particular interest (Lismer et al., 2020, 2021). Dietary exposure changes the H3K4me3 profile in sperm and those changes affect their offspring’s phenotype (Lismer et al., 2021). The potential role of the active H3K4me3 in transgenerational inheritance has also been reported in *Caenorhabditis elegans* (Greer et al., 2011). Therefore, the alterations in sperm H3K4me3 could be an evolutionary conserved pathway to achieve transgenerational inheritance. Since loci-retained histone modification could be environmentally altered (Terashima et al., 2015; Lismer et al., 2021), environmental exposure to toxins may affect the imprinting status and affect the health of offspring. Understanding whether altered H3K4me3 in sperm dysregulates imprinted gene expression is another critical question for the field to resolve.

This study identified 13 new transient PEGs during zygotic activation. Seven out of the 13 genes had ERV family motifs, such as ERVL-MalR and ERVK within +2.5 kb of their transcription start site. Given that H3K4me3 is already present at the genes before the zygotic genome activation, the ERV motifs are not likely to be involved in establishing the heritable chromatin status of the paternal allele, which was the focus of this study. It is possible, however, that the ERV motifs may help in recruiting transcription machinery to the subset of PEGs at the onset of zygotic genome activation. Since expression of an endogenous retroviral element, MERVL, is known to be activated by the DUX transcription factor (Schulz and Harrison, 2019), it is possible that DUX activates the transient PEGs during zygotic genome activation. Identification of molecular machinery that regulates the transient PEGs during zygotic genome activation could possibly elucidate the links between ERV motif and their expression. However, this is beyond the scope of this study.

Eight out of the 13 new transient PEGs were associated with paternal H3K4me3 before (in sperm) and after fertilization. Using parental SNP information, we confirmed that seven out of eight new transient PEGs developed biallelic expression as embryogenesis proceeded, suggesting that if they have a function, it can only be in the early stages of preimplantation development. For example, one of the new transient PEGs identified in this study, *Eva1a*, is known to regulate cell autophagy (Zhang et al., 2017). Autophagy is considered as an essential process for the active elimination of unnecessary suppressors of the zygotic gene program within oocytes (Tsukamoto et al., 2008). Paternal Eva1a may, therefore, contribute to the process of zygotic genome activation by regulating autophagy. A newly identified transient PEG, *Prkcz*, lost imprinted gene expression after the eight-cell stage. The pattern and order of cell division between the two- and four-cell stages affect both fate and developmental potential of the resulting cells (Ajduk and Zernicka-Goetz, 2016), and Prkcz is known to be involved in the cell polarity pathway (Huang and Muthuswamy, 2010). Prkcz might, therefore, be involved in the cleavage process by defining planes of cell division. The newly identified transient PEG could be involved in the progress of preimplantation development. A newly described transient PEG, *Cry2*, also lost its imprinted gene expression after the eight-cell stage. Cry2 is known as a circadian repressor, and the circadian clock can interact with the cell cycle dynamically (Chan et al., 2020). It is possible that Cry2 could be involved in the regulation of the order of cell divisions from the two- to four-cell stage. Another new transient PEG, *Atxn10*, is known to be involved in cytokinesis because knockdown of *ATXN10* causes cytokinesis defects and multinucleation in HeLa cells (Li et al., 2011) and *Atxn10* null mice are embryonic lethal at early postimplantation stages (Wakamiya et al., 2006). Normal embryonic development requires contributions from both parents (Barton et al., 1984); a phenomenon that emphasizes the important developmental role of imprinting. In this context, the finding in this study that some of the transient PEGs in the early mouse embryo are known regulators of cell development is particularly significant. This finding may provide support for the hypothesis that imprinting has its evolutionary origins in the prevention of potentially pathological parthenogenic development and trophoblastic disease [see the ovarian timebomb hypothesis (Weisstein et al., 2002)]. Further identification and characterization of transient PEGs in subsequent studies will help to clarify why there are genes expressed in a parent-of-origin-specific manner in early development.

## Data Availability

The original contributions presented in the study are included in the article/Supplementary Material. Further inquiries can be directed to the corresponding author.
