# Supplementary material for: Presence of H3K4me3 on Paternally Expressed Genes of the Paternal Genome From Sperm to Implantation
Source: Front Cell Dev Biol. 2022 Mar 10;10:838684. doi: 10.3389/fcell.2022.838684 (PMC8960379; doi:10.3389/fcell.2022.838684)
Supplement: Supplementary file 1 [file DataSheet1.docx]

Supplementary Material


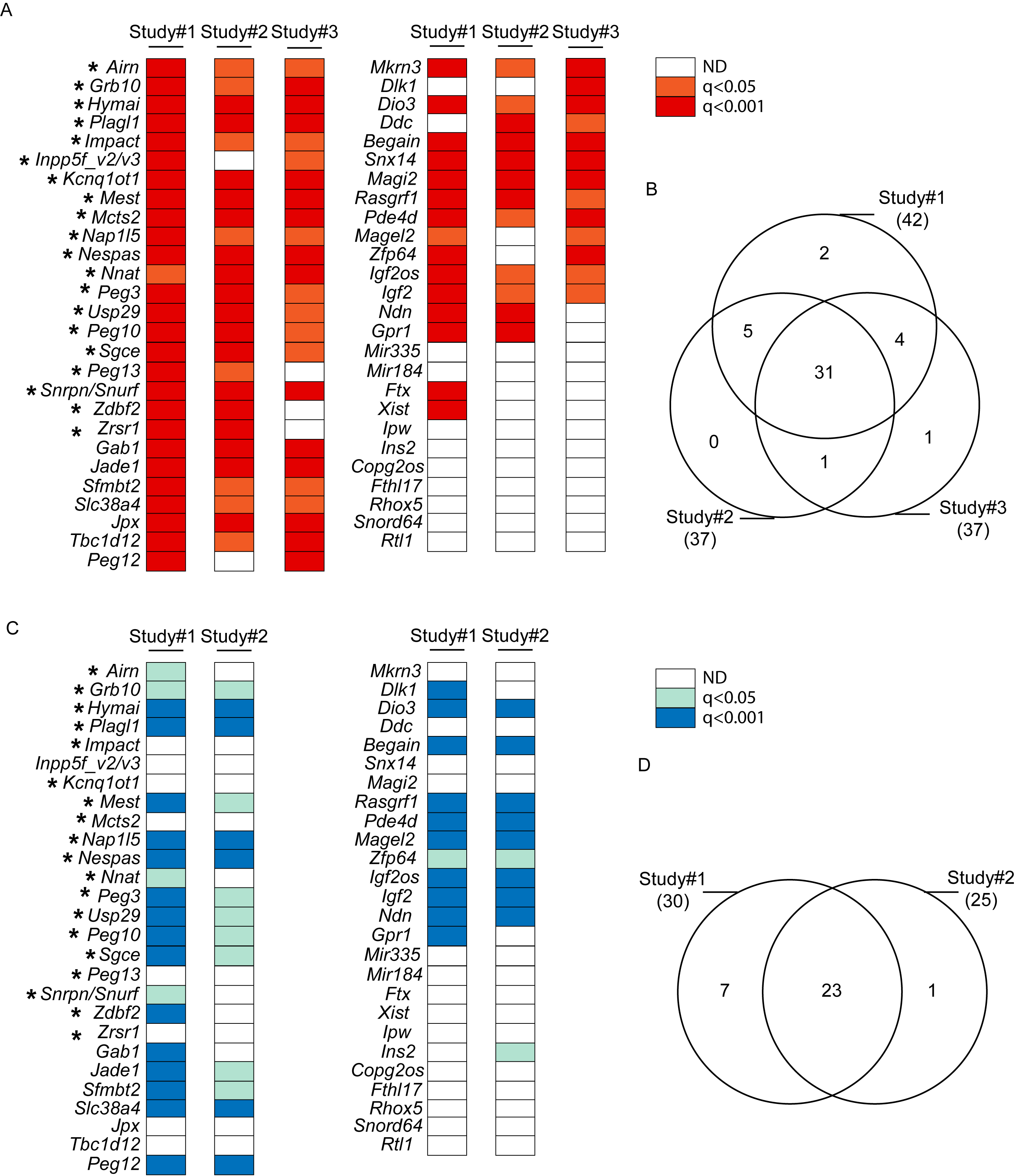


**Supplementary Figure S1.** **Sperm H3K4me3 and H3K27me3 status at the known PEGs. A.** Sperm H3K4me3 at PEGs in the three different datasets examined in this study. Study #1, Study #2 and Study #3 represent datasets in Erkek et al (2013), Zhang et al (2016) and Yamaguchi et al (2018), respectively. **B.** Comparison of H3K4me3 associated paternally expressed gene between data sets. The Venn diagrams show the number of genes common between datasets. **C.** Sperm H3K27me3 at PEGs in the three different datasets examined in this study. Study #1 and Study #2 represent datasets in Erkek et al (2013) and Zheng et al (2016), respectively. **D.** Comparison of H3K27me3 associated paternally expressed gene between data sets. The Venn diagrams show the number of genes common between datasets. All q-values are based on MACS2 peak calling.

**
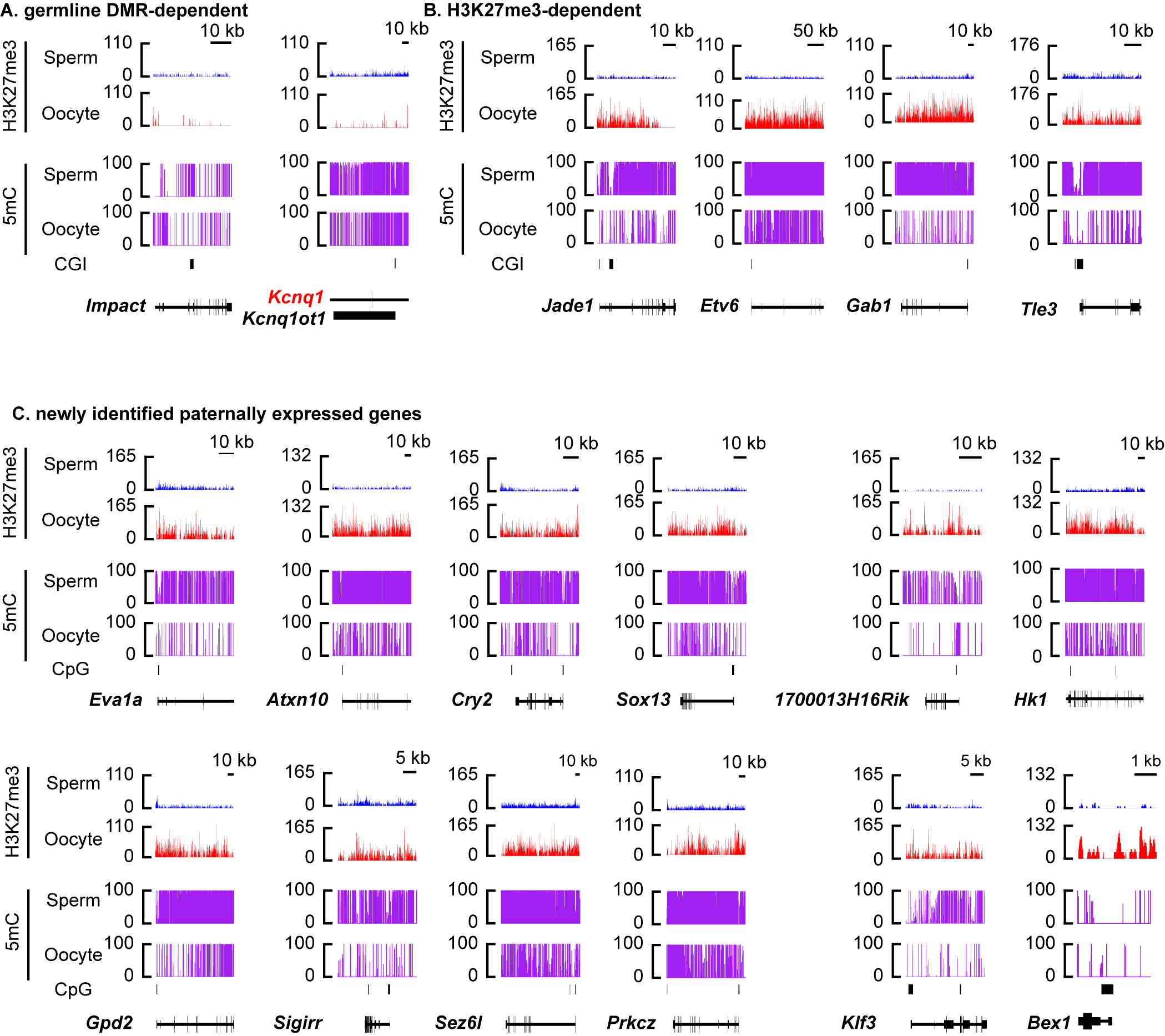
**

**Supplementary Figure S2.** **DNA methylation and H3K27me3 status of the new paternally expressed genes in sperm and MII stage oocyte**. **A.** Germline DNA methylation dependent imprinted genes, Impact and Kcnq1ot1. **B.** H3K27me3-dependent imprinted genes. **C.** Newly identified paternally expressed genes. There was no maternally enriched DNA methylation on promotor CpG islands at the new paternally expressed genes except *1700013H16Rik* and *Bex1*. All of the 12 genes showed enrichment of H3K27me3 in oocyte.

**Supplementary Table S1. A detailed list of data sets used for this study.**

| **Stage** | **Type** | **Sample** | **ID** | **Accession number** |
| --- | --- | --- | --- | --- |
| Round spermatid | ChIP-seq | H3K4me3_ChIPSeq_ST | GSM1202707 | GSE49624 |
|  |  | H3K27me3_ChIPSeq_ST | GSM1202710 |  |
|  |  | Input_ChIPSeq_ST | GSM1202725 |  |
| Sperm | ChIP-seq | Sperm Input | DRR124337 | DRX117177 |
|  |  | Sperm H3K4me3 | DRR124333 |  |
| Sperm | ChIP-seq | Sperm sonicated genomic DNA | GSM1046836 | GSE42629 |
|  |  | Sperm H3K4me3 | SM1046833 |  |
|  |  | Sperm H3K27me3 | GSM1046835 |  |
| Sperm | ChIP-seq | Sperm H3K4me3_rep1 | GSM2101163 | GSE71434 |
| MII_oocyte |  | MII_oocyte_H3K4me3_rep1 | GSM1845262 |  |
|  |  | MII_oocyte_H3K4me3_rep2 | GSM1845263 |  |
| Zygote (PN3) |  | Zygote_PN3_H3K4me3_Maternal pronucleus | GSM2101161 |  |
|  |  | Zygote_PN3_H3K4me3_Paternal pronucleus | GSM2101162 |  |
| Zygote (PN5) |  | Zygote_PN5_H3K4me3_rep1 | GSM1845264 |  |
|  |  | Zygote_PN5_H3K4me3_rep2 | GSM1845265 |  |
| 2-cell (Early) |  | 2cell_early_H3K4me3_rep1 | GSM1845266 |  |
|  |  | 2cell_early_H3K4me3_rep2 | GSM1845267 |  |
| 2-cell (Late) |  | 2cell_late_H3K4me3_rep1 | GSM1845268 |  |
|  |  | 2cell_late_H3K4me3_rep2 | GSM1845269 |  |
| 4-cell |  | 4cell_H3K4me3_rep1 | GSM1845270 |  |
|  |  | 4cell_H3K4me3_rep2 | GSM1845271 |  |
| 8-cell |  | 8cell_H3K4me3_rep1 | GSM1845272 |  |
|  |  | 8cell_H3K4me3_rep2 | GSM1845273 |  |
| Blastocyst  (ICM) |  | ICM_H3K4me3_rep1 | GSM1845274 |  |
|  |  | ICM_H3K4me3_rep2 | GSM1845275 |  |
| MII_oocyte | RNA-seq | MII_oocyte_rep1 | GSM1845295 |  |
|  |  | MII_oocyte_rep2 | GSM1845296 |  |
| Zygote |  | Zygote_PN5_rep1 | GSM1845297 |  |
|  |  | Zygote_PN5_rep2 | GSM1845298 |  |
| 2-cell (Early) |  | 2cell_early_rep1 | GSM1845299 |  |
|  |  | 2cell_early_rep2 | GSM1845300 |  |
| 2-cell (Late) |  | 2cell_late_rep1 | GSM1845301 |  |
|  |  | 2cell_late_rep2 | GSM1845302 |  |
| 4-cell |  | 4cell_rep1 | GSM1845303 |  |
|  |  | 4cell_rep2 | GSM1845304 |  |
| 8-cell |  | 8cell_rep1 | GSM1845305 |  |
|  |  | 8cell_rep2 | GSM1845306 |  |
| Blastocyst  (ICM) |  | ICM_rep1 | GSM1845307 |  |
|  |  | ICM_rep2 | GSM1845308 |  |
| 2-cell (Early) | RNA-seq | C57 x PWK 2cell early rep1 | SRR8626402 | GSE127106 |
|  |  | C57 x PWK 2cell early rep2 | SRR8626403 |  |
|  |  | PWK x C57 2cell early rep1 | SRR8626412 |  |
|  |  | PWK x C57 2cell early rep2 | SRR8626413 |  |
| 2-cell (Late) |  | C57 x PWK 2cell late rep1 | SRR8626404 |  |
|  |  | C57 x PWK 2cell late rep2 | SRR8626405 |  |
|  |  | PWK x C57 2cell late rep1 | SRR8626414 |  |
|  |  | PWK x C57 2cell late rep2 | SRR8626415 |  |
| 4-cell |  | C57 x PWK 4cell late rep1 | SRR8626408 |  |
|  |  | C57 x PWK 4cell late rep2 | SRR8626409 |  |
|  |  | PWK x C57 4cell late rep1 | SRR8626418 |  |
|  |  | PWK x C57 4cell late rep2 | SRR8626419 |  |
| MII_oocytes | WGBS | MII_oocytes_bulk_WGBS | SRR1248456 | GSE56879 |
| Sperm | ChIP-seq | PWK sperm K27Me3 | GSM2041066 | GSE76687 |
| MII_oocytes |  | MII oocyte K27Me3 rep1 |  |  |
|  |  | MII oocyte K27Me3 rep2 |  |  |
| Androgenotes  (2-cell) | RNA-seq | 2C_AG_rep1 | GSM2433399 | GSE92605 |
|  |  | 2C_AG_rep2 | GSM2433400 |  |
| Parthenogenotes  (2-cell) |  | 2-Cell Parthenogenote rep1 | GSM2433401 |  |
|  |  | 2-Cell Parthenogenote rep1 | GSM2433402 |  |
| Amanitin-treated  2-cell |  | Amanitin_2C_rep1 | GSM2433403 |  |
|  |  | Amanitin_2C_rep2 | GSM2433404 |  |
